# New Charter of the College of Surgeons

**Published:** 1844-01

**Authors:** 


### PART FOURTH.

### Medical Intelligence.

### New Charter of the College of Surgeons.

The following is an abstract of the provisions of the new charter of the College of Surgeons, freed from legal verbiage and technicalities. We append a few remarks which naturally suggest themselves on the examination of this important document.

1. The name of the College is changed to that of The Royal College of Surgeons of England.

2. The future members are to consist of two classes, Members and Fellows.

3. Members are to be admitted precisely as heretofore, after an ordinary examination, and at any age not less than twenty, and on paying the usual fee, (at present twenty guineas.)

4. Persons are admissible as Fellows after passing a special examination, and at any age not less than twenty-five. It is not necessary that the candidate for the Fellowship should previously pass the examination for a member; but persons having become members may present themselves for examination as fellows, on attaining the proper age.

5. The scholastic educational requirements and the nature of the examinations for candidates for the Membership and Fellowship, respectively, are not defined in the Charter.

6. There is no other restriction as to the attainment of the Fellowship, except those of age and passing the appointed examination. [Fellows consequently may practise pharmacy and midwifery at will.]

7. The number of Members and Fellows is unlimited.

8. Fellows have no special privileges beyond Members, except in having votes for the Members of the Council, and in being themselves eligible as Members.

9. The amount of fee to be paid by Fellows, on admission, is left to be fixed by the Council, but cannot exceed thirty guineas over and above the stamp duty on the diploma. [Whatever be the amount of fee fixed on for the fellowship, it is presumed that the sum previously paid by members for their diploma will be deducted from it.]

10. The Council is henceforth to consist of twenty-four Members (instead of twenty-one as at present,) to be elected from the Fellows by the Fellows.

11. All Fellows are eligible for the Council, except such as actually practise, or during the preceding five years have practised pharmacy or midwifery, or live beyond five miles from the London Post-Office.

12. Three Members are to retire from the Council every year, and their places to be filled up from the Fellows: the retiring members to be immediately re-eligible.

13. The election of Councillors is to be by ballot of the Fellows present at the meeting, fifteen to be a quorum, and the election to be decided by a simple majority of votes.

14. At the election of Councillors, Fellows must be proposed in the order of their seniority on the list; and every Fellow so proposed on two different occasions and not elected, shall not be again eligible.

15. The members of the Council in office at the time of granting the Charter are to continue such for life without re-election.

16. Until the Life-Members have all died off or retired, the annual supply of three new Members to the Council is to be effected by the retirement of the Members last elected.

17. The Examiners of candidates for the Membership and Fellowship are to be ten in number. They are to be chosen by the Council from the Fellows whether at the time members of the Council or not. They are to hold office during the pleasure of the Council. Six examiners are to be a quorum for examination. [The present Examiners are to continue for life.]

18. The President and the two Vice Presidents are to be chosen by the Council from among the Members of the Council, whether Examiners or not.

19. The new Charter confirms all the powers of the old, and bestows no additional powers, except such as are stated in the foregoing extract.

20. All by-laws and ordinances of the Council must be approved of by the Secretary of State before they are valid.

21. The Charter grants to the old Council the provisional power of nominating, and commands it to nominate, without examination or the payment of any fee, a certain number of Fellows from among the existing Members of the College, namely, not less than 250, nor more than 300, within three months from the date of the Charter, that is before the 14th of December, 1843 ; and authorizes it, but does not order it, to nominate other Members (the number not specified) to be Fellows, within the period of nine months after the 14th of December. After the expiration of the twelve months from the date of the Charter, Fellows can only be elected after examination and the payment of a fee as provided by the Charter.

22. The Council are ordered to keep a register of all the FELLOWS in the order of their seniority for the inspection of Fellows and Members. [This register, it is presumed, will contain also a list of MEMBERS, with the place of residence of both Fellows and Members, and will be published annually for their use.]

The first and most important remark that suggests itself in reference to this Charter, is, that its whole purport is to regulate the College as a simple corporate body, and that it does not confer on it one atom of power or a single privilege in relation to the profession. The new College, like the old, can give no licence to practise, possesses no control whatever over any class of practitioners, and can oblige no one to enter its ranks either as a Member or Fellow. All its privileges consist in its power to bestow a title on those who choose to seek it, according to certain regulations. The organization and government of the MEDICAL PROFESSION, therefore, are not at all interfered with by the provisions of the Charter, and await the enactment of the MEDICAL BILL, so long in preparation by Sir James Graham. It is, no doubt, unfortunate that the granting of this Charter was not deferred until after the passing of the bill; as the provisions of the Charter—which can only be regarded as a mere subordinate matter of detail—may be found to hamper the more important provisions of the general measure. The difficulty will be increased if the Physicians' Charter is also granted previously to the enactment of the Medical Bill. We hope, however, that although it will be more difficult to frame a liberal general law in perfect harmony with these Charters than it would have been to frame Charters in harmony with it, yet that the difficulty is not insuperable. Should this, indeed, prove to be the case, we doubt not that the author of the bill will not hesitate to get both Charters modified, rather than endanger the soundness of a measure on which the future welfare and prosperity of the whole medical profession will probably depend. Our object, however, at present being merely to offer a few comments on the new Charter, as a code of laws for regulating one of the most important corporate societies in the profession, we shall leave all consideration of the general subject until another occasion.

It will not, we presume, be doubted by any calm and impartial judge, that this Charter is a great improvement on the old. Some of its provisions certainly might have been improved; but, looking at it as it stands, we think the profession owes a debt of gratitude to Sir James Graham for insisting (as we believe he did) on its more liberal clauses ; while it is, in many respects, creditable to the judgment of those Members of the College who were mainly instrumental in obtaining its enactment.

No man of experience need be told that it could be no easy task to bring a body of men, however honorable, in the actual possession of exclusive rights of long standing, of power and of property, and responsible to no one, to forego, in a great measure, their dearest privileges. And it were well that when we criticise the proceedings of such persons, we should reflect what might have been our own feelings, views, and conduct in similar circumstances. Examining the Charter in this spirit we must regard its provisions, on the whole, as good ; and we think we may confidently expect from those who framed it, yet further improvements in its more liberal parts, if it should be found, in the working, to be injuriously restrictive of the legitimate rights and free action of the new constituency.

By the new Charter the system of monopoly and self-election is for ever abolished ; and it remains entirely with the future Members of the College whether the Council shall henceforth be such as they deem fit to represent and rule them. As by the new provisions, every member may claim to be admitted a Fellow on attaining the age of twenty-five; and as the election of the Council is left entirely in the hands of the Fellows, no apprehension need be entertained that the appointment of Councillors will be brought about by unfair means, or, consequently, that improper persons will be elected. The examination for the Fellowship will, of course, be of such a nature as any well-informed surgeon can pass—no Council would dare to make it unnecessarily severe—it cannot, therefore, be doubted but that the great majority of future Members will become Fellows, and thus a body of electors will always exist too large to be corrupted or seduced or "managed" in any way. Most important advantages will result from making the admission to the Fellowship through the door of a higher examination, and at a period subsequent to the ordinary termination of school studies. The men desirous of this honour—and, as we have said, we believe most members will be so—will take care that in the first instance they *study up to the mark* of the Fellowship; and that should they at first take the inferior degree of Member, they will *keep up* the knowledge then obtained, or even continue to improve it, until they reach the age for claiming the higher title. Had we been framing the Charter, we should have made the examination for the membership a strict one, and the only one, leaving admission to the Fellowship contingent on the attainment of a certain standing as Member—say seven or ten years—and the payment of a moderate fee. We, however, do not quarrel much with the present arrangement, and see in it some advantages over the other, the most important of which is that just stated. A more serious objection to the new Charter in the eyes of many will probably be the exclusion from the Council of practitioners of pharmacy and midwifery. In some respects we consider this as an important defect, more especially as regards the practitioners of midwifery. Although well aware that the progress of reform is not yet sufficiently advanced to justify any measure that would virtually forbid surgeons to practise pharmacy (that is, to send out their own medicines), we are still glad to see any enactment that tends to abolish this most impolitic, and, we may say degrading association of trade with science. And we entertain no doubt whatever that the clause excluding practitioners in pharmacy from the Council of the College, will eventually have a powerful influence in abating this crying evil. Unfortunately, however, the coupling of midwifery with pharmacy as joint causes of exclusion, will interfere most materially with the progress of this reform. General practitioners may, without difficulty, cease to supply their patients with drugs; but we cannot see how they can cease to attend cases of midwifery without such a sacrifice of their interests as must more than counterbalance even the honours and advantage of a seat in the Council. In our zeal for the abolition of the trade in physic, we would have almost consented to the exclusion of practitioners of pharmacy even from the Fellowship, provided this obnoxious and impolitic exclusion of practitioners in midwifery from the Council had been abandoned. After all, however, this can only be regarded as a minor evil; and sinks into insignificance when viewed in conjunction with the real and substantial advantages conferred by the Charter. We cannot doubt but that there will always be found in London and its vicinity enow of men in the class of pure surgeons (including many who have ceased to practise pharmacy and midwifery) from among whom the Fellows—that is, THE GENERAL PRACTITIONERS (*for the vast majority of Fellows will eventually be general practitioners*)—may choose fitting representatives and officers. It even will lie with themselves, hereafter, to obtain such modifications in the Charter as they may deem more to their own honour and advantage. Every general practitioner may become a Fellow if he please; and every Fellow may, even by the present Charter, render himself eligible for the Council: and it will go hard, if, with such potential vantage-ground, the majority should not be able to obtain the virtual, if not the ostensible supremacy in the direction of their own affairs.

The prescribed mode of electing members of the Council (see paragraph 14 of our abstract) seems to us, also, if not a positive defect, certainly of doubtful propriety. It, no doubt, obtained admission into the new Charter because it formed a prominent feature in the old,—of which, indeed, it is an improvement. The obvious and natural mode of proceeding, as it appears to us, would have been—to limit the eligibility to be members of Council, to Fellows of a certain standing—say ten years—and then leave the selection to be made from this class, without any restriction as to individual seniority. And we cannot but apprehend that the reasonable desire on the part of the Fellows, to choose the men they may deem best qualified for the office of Councillors, may, in the working of this part of the measure, lead to results seriously affecting the harmony and respectability of the institution. We doubt not, however, that the more liberal members of the Council will take care, by great reserve in proposing the immediate re-election of retiring Members, not to compromise the rights or just expectations of those placed lower on the list. In this manner we think the clause in question may, in a great measure, be deprived of the danger of which it appears to us to be at least a possible source. With regard to this clause, however, as well as to others which may seem objectionable, we doubt not that the new Fellows will have sufficient judgment and discretion not to condemn it or them absolutely, until their value has been proved in actual practice, and on more than one occasion; as it will be much more for their interests to put up with inconveniences or evils for a few years, and then to have them permanently remedied, than to run the risk, by a hasty decision, of only exchanging one blemish for another. There was never yet a code of laws devised for the government of any institution which did not call for and receive subsequent alteration and improvement; and we have no right to expect that the new code of the College of Surgeons should differ in this respect from all its predecessors.

Viewed in relation to the great question of the nature of the Charter itself and its future working, it is a matter comparatively of trifling importance, whether the retained Life Members of the Council are well qualified or not, or whether in selecting the new Fellows they have exercised their privilege with impartiality and judgment. The evil, if it existed, would be soon abated by the great redressor of wrongs, Time. Alas, in the course of twenty or thirty years, where will be the great majority of the Life Members, of the Selected Fellows, and of Us who may be criticising them and their proceedings? Ere the expiration of that period, the great body of the Fellows will be those who had attained their station by right, and the Members of the Council will be of their own choosing, and directly or indirectly responsible to them. We are, therefore, little disposed to give heed to the short-sighted critics who may be able to show us some imperfections in this part of the arrangements. But the fact we sincerely believe to be—that the present Members of the Council will, with the new infusion of the Annual Three, constitute a good Council; and we have every reason to believe that they have exercised their privileges of selecting the new Fellows with sound discretion and impartiality. That the list should give universal satisfaction is not to be expected; but knowing, as we do, the men who are said to have been principally active in forming it, we shall be surprised if the names contained in it do not obtain very general assent from the profession.

We have reason to believe that the Council, in selecting the new Fellows, have had chiefly in view individuals coming under one or other of the following denominations:

1. Members of the present Council.
2. Surgeons of recognized hospitals.
3. Retired surgeons of recognized hospitals.
4. Practitioners in London who were regarded as eligible to the Council under the old system.
5. Medical officers of the army, navy, and East India Company's service, specially recommended by the heads of their department.
6. Practitioners in the country, not hospital surgeons, but having a high surgical reputation in their respective districts, and being of a certain standing in the College.
7. Other Members of the College, not included under the foregoing heads, but distinguished for their scientific labours or high professional eminence.

N.B. The first list of Fellows is limited to 300, and includes no one whose diploma as Member dates since March 1837. The supplementary list, to be published within twelve months from the date of the Charter, will contain the juniors and some others whose names were omitted for want of room in the first.

